# Complex Congenital Anomalies: A Case Report of Left Pulmonary Hypoplasia, Absent Left Pulmonary Artery, and Congenital Heart Disease in an 11-Month-Old Male Child

**DOI:** 10.7759/cureus.63430

**Published:** 2024-06-28

**Authors:** Naramreddy Sudheesh Reddy, Ashish Varma, Keta Vagha, Vaibhav Raut, Chaitanya Kumar Javvaji, Sai Bhavani Manchineni, Anirudh Kommareddy, Jayant D Vagha, Siddhartha Murhekar

**Affiliations:** 1 Pediatrics, Jawaharlal Nehru Medical College, Datta Meghe Institute of Higher Education and Research, Wardha, IND; 2 Cardiology, Jawaharlal Nehru Medical College, Datta Meghe Institute of Higher Education and Research, Wardha, IND; 3 Trauma and Orthopaedics, East Kent Hospitals University NHS Foundation Trust, Canterbury, GBR

**Keywords:** clinical cardiology, congenital cardiac anomalies, pulmonary artery, ventricular septal defect (vsd), pulmonary hypoplasia

## Abstract

A rare disorder called pulmonary hypoplasia is characterized by inadequate lung development, which frequently results in respiratory dysfunction and other related abnormalities. We present a case of an 11-month-old male child with left lung hypoplasia, absent left pulmonary artery, and ventricular septal defect (VSD). The child exhibited symptoms of cough and cold, with a history of recurrent respiratory tract infections since birth. Cardiovascular examination revealed a pan systolic murmur consistent with VSD, while respiratory examination indicated decreased air entry on the left side. Imaging studies confirmed the absence of the left pulmonary artery and left lung hypoplasia. Despite recommendations for VSD surgery, the child's parents declined surgical intervention, leading to discharge against medical advice. This case highlights the challenges in managing pulmonary hypoplasia, especially when accompanied by complex congenital heart defects, and underscores the importance of multidisciplinary care and parental involvement in decision-making.

## Introduction

A relatively uncommon medical disorder called pulmonary hypoplasia causes the lung tissues to underdevelop, leading to incomplete lung growth that can have a significant effect on a child's development [1]. Because there are fewer airways and alveoli in this situation, there is generally insufficient gas exchange and respiratory impairment [2]. In the general population, the incidence of pulmonary hypoplasia ranges from 0.9 to 1.1 per 1000 live births, with an approximate value of 1.4 per 1000 births [3]. Primary pulmonary hypoplasia refers to defective lung development inherent to the condition, whereas secondary pulmonary hypoplasia refers to lung development hampered by another abnormality.

Secondary pulmonary hypoplasia is frequently caused by oligohydramnios, which can be caused by kidney abnormalities, placental defects, or intrauterine development limitation [4]. Since Frantzel's 1868 description of the absence of a primary pulmonary artery branch, many cases with and without intracardiac anomalies have been recorded [5]. Congenital cardiac conditions are frequently linked to pulmonary hypoplasia [6]. In this case report, we describe an 11-month-old child who has been diagnosed with hypoplasia of the left lung, along with an absent left pulmonary artery and ventricular septal defect (VSD).

## Case presentation

An 11-month-old male child presented to our hospital with a history of cough and cold for the past 10 days. The child was healthy until 10 days ago when he developed a dry cough, which was alleviated with medication. The child has a history of recurrent respiratory tract infections and exhibits a suck-rest-suck feeding cycle along with forehead sweating. The child was born via normal vaginal delivery. At birth, the child cried immediately and was admitted to the neonatal intensive care unit due to respiratory distress. He was initially managed with continuous positive airway pressure and then gradually weaned to nasal prongs and room air. During this neonatal period, he was diagnosed with VSD and advised for follow-up.

The child presented with complaints of cough and cold and was admitted to the ward. Upon admission, vital signs were respiratory rate of 58 cycles per minute, oxygen saturation of 97% on room air, heart rate of 92 beats per minute, and blood pressure of 86/50 mmHg. Cardiovascular examination revealed a thrill, parasternal heave, and a pan-systolic murmur. Respiratory examination indicated decreased air entry on the left side, while other systemic examinations were normal. Laboratory investigations showed elevated total leucocyte count and other laboratory investigations were within normal limits, as shown in Table 1.

**Table 1:** Laboratory investigations of the child PT: prothrombin time, INR: international normalized ratio, APTT: activated partial thromboplastin time

Laboratory investigations	Day 1	Biological reference range
Hemoglobin	11.4	13-15 g/dl
Total leucocyte count	14500	4000-11000/ cumm
Platelet	1,70,000	1,50,000-4,50,000/cumm
Urea	16	9-20 mg/dl
Creatinine	0.5	0.6-1.2 mg/dl
Sodium	136	137-145 mmol/l
Potassium	3.8	3.5-5.1 mmol/l
Alkaline phosphatase	130	38-126 unit/l
Alanine transaminase	42	<50 U/l
Aspartate transaminase	50	17-59 U/L
Total protein	7.0	6.3-8.2 gm/dl
Albumin	3.8	3.5-5 gm/dl
Total bilirubin	1.0	0.2-1.3 mg/dl
Unconjugated biliribin	0.2	0-0.3 mg/dl
Conjugated bilirubin	0.8	0-1.1 mg/dl
Globulin	3.2	2.3-3.5 mg/dl
APTT	30.1	29.5 Control
PT	12.2	11.9 Control
INR	1.1	0.8-1.2

**Figure 1:**
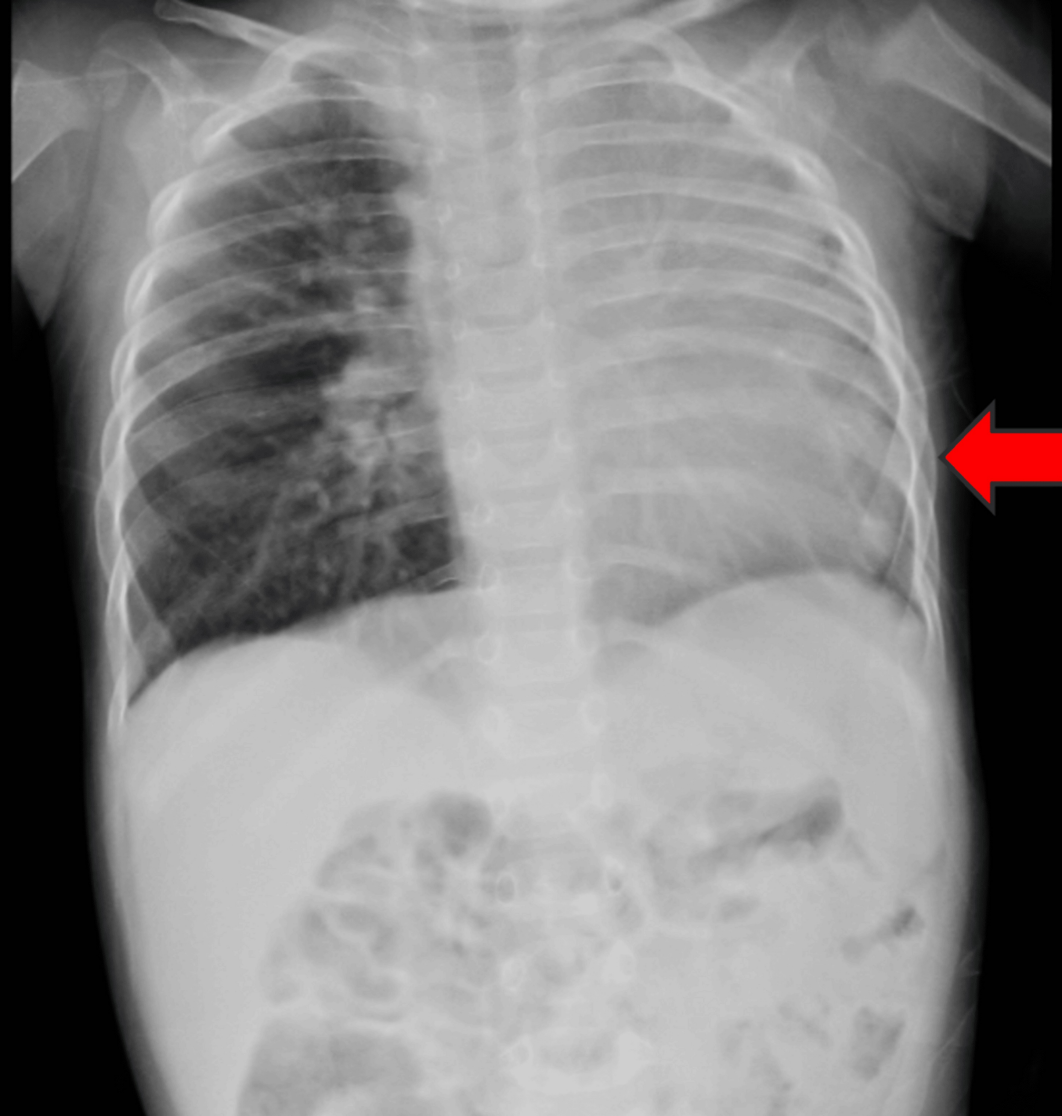
Chest X-ray revealing hypoplasia of the left lung (red arrow)

The child was started on furosemide (1 mg/kg/day), vitamin D3 drops, intravenous amoxicillin (100 mg/kg/day), and nebulization with levosalbutamol (0.05 mg/kg/dose). A chest X-ray revealed hypoplasia of the left lung (Figure 1).

**Figure 2:**
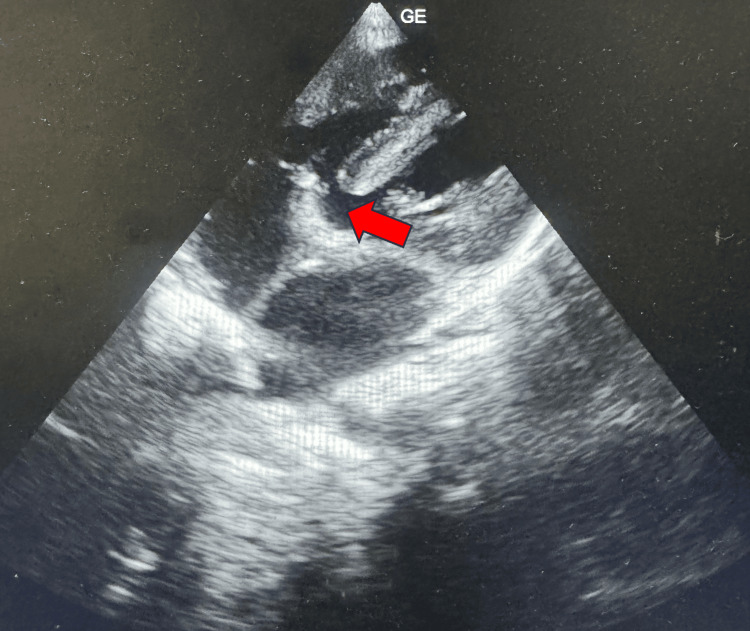
2D echocardiography showing ventricular septal defect (red arrow)

Subsequent 2D echocardiography confirmed the presence of a ventricular septal defect and an absent left pulmonary artery (Figure 2).

**Figure 3:**
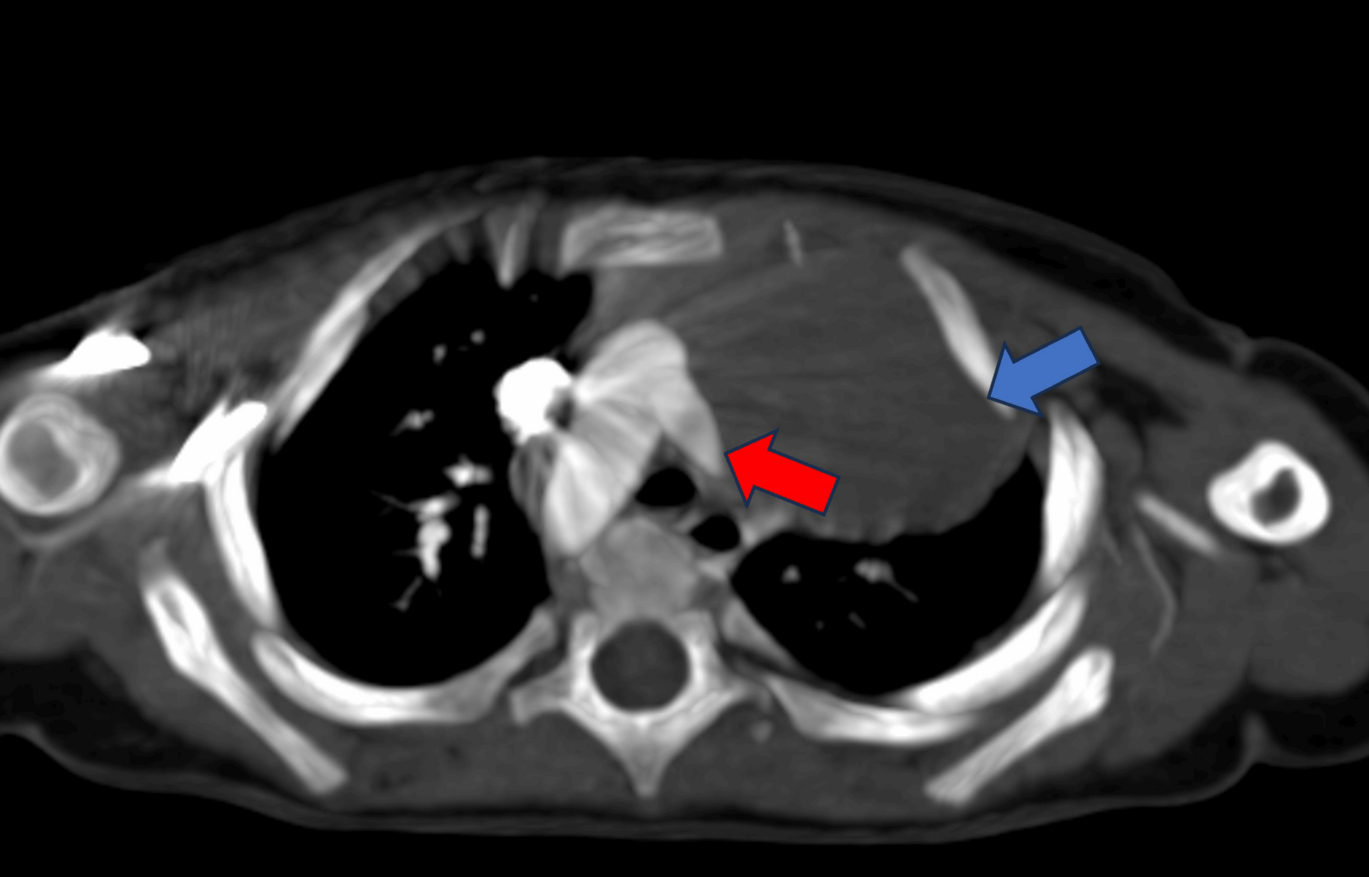
Computed tomography pulmonary angiography revealed an absent left pulmonary artery (red arrow) and left lung hypoplasia (blue arrow)

Computed tomography pulmonary angiography revealed an absent left pulmonary artery and left lung hypoplasia (Figure 3).

Surgical correction of the VSD was recommended. However, the child's parents declined the surgical intervention, leading to the child being discharged against medical advice. Written consent was obtained from the parents, authorizing the disclosure of images in the case report.

## Discussion

A significant cause of neonatal infant death, pulmonary hypoplasia is prevalent during the perinatal stage [7]. Usually, pulmonary hypoplasia is the outcome of an underlying issue. Normal fetal lung requires fluids at positive pressure, normal breathing patterns, a normal thoracic cavity, and a normal volume of amniotic fluid for healthy lung development in utero, even though the precise cause of pulmonary hypoplasia is unknown.

Various categories of underlying disorders can lead to pulmonary hypoplasia. Chest space-occupying lesions, including pleural effusions and displaced abdominal organs in congenital diaphragmatic hernia, can aggravate the condition. Another group includes abnormalities in the chest wall that cause the thoracic cavity to become smaller, such as kyphoscoliosis. Another critical factor is oligohydramnios, which can occur from renal tissue failure, chronic premature rupture of the membranes, or urine outflow obstruction. Furthermore, pulmonary hypoplasia may develop as a result of neuromuscular abnormalities that impede normal fetal breathing movements [8].

According to Wigglesworth et al., hypoplastic lungs caused by oligohydramnios are morphologically and biochemically immature for the lung's gestational age [9]. These circumstances lead to poor lung phospholipid concentrations, insufficient epithelial development, insufficient growth and maturity of the acinus's peripheral segments, and delays in the blood-air barrier's formation. On the other hand, hypoplastic lungs, for reasons other than oligohydramnios, usually have a structure suitable for the gestational period. The investigators discovered that the incapacity to retain embryonic lung fluids may be directly linked to the maturation arrest observed in pulmonary hypoplasia caused by oligohydramnios [9].

However, the maturity or shape of hypoplastic lungs linked to renal agenesis or dysplasia does not differ from those linked to other illnesses, according to other research [10]. The lungs show normal bronchial development in pulmonary hypoplasia caused by congenital acinar dysplasia; nonetheless, each lobule comprises terminal bronchiole-like structures with many cystic outpouchings surrounded with bronchial-type epithelium and lacking alveoli. This disorder seems to be a fundamental malformation of the lungs [11].

The majority of newborns with bilateral lung abnormalities or without any pulmonary development typically do not have long-term survival [6]. If there is sufficient compensatory activity, patients with unilateral pulmonary agenesis or pulmonary aplasia may live [12]. Nevertheless, congenital heart disease, tracheoesophageal atresia, spinal cord deformities, and severe renal failure are among the additional severe anomalies frequently present in these patients. Severe symptoms such as dyspnea, refractory respiratory infections, and feeding difficulties are commonly associated with these diseases. As a result, over half of these instances do not make it past infancy [13].

The degree of deformities present in pulmonary hypoplasia cases and any coexisting abnormalities are related to the severity of the disease. In infants, patients with modest severity may not show any symptoms but may later develop refractory respiratory infections and worsening of the illness. Severe cases frequently exhibit diminished respiratory movement and noises, dyspnea, cyanosis, and respiratory failure, as well as a shift in heart sounds to the affected side [14].

The most common cardiac conditions associated with pulmonary hypoplasia include Ebstein's abnormalities, pulmonary stenosis, scimitar syndrome, hypoplastic right heart, and tetralogy of Fallot (TOF) [15]. It is yet unknown what caused these ipsilateral congenital abnormalities. The level of hypoplasia and related anomalies dictate the degree of respiratory impairment and the clinical presentation. As of right now, there are no proven treatments for pulmonary hypoplasia. In a 2005 patient with right lung hypoplasia coupled with both TOF and TAPVC, Festa et al. [16] successfully completed a modified Glenn anastomosis and corrected the anomalous pulmonary venous connection (TAPVC). In 2013, Patnaik et al. reported a case of VSD and congenital pulmonary artery aneurysm [17].

Imaging data that may aid the diagnosis include enhanced and contrast CT scans, thoracic X-rays, MRA, angiography, pulmonary function tests, and bronchoscopes. To support the typical cases, the thoracic X-ray may demonstrate small size in the involved lung, decline in pulmonary marking, diaphragm elevation, and deviation of the mediastinum to included areas. Differentiating pulmonary hypoplasia from bronchial asthma, foreign body inhalation-induced pulmonary atelectasis, and newborn pneumonia is crucial. In our instance, there was a decline in left lung lucency and a decrease in air entry. Lobar pneumonia in the left lung was suspected at that moment.

The symptoms and results of the chest X-ray for atelectasis brought on by a foreign body in the bronchus were comparable to those of pulmonary hypoplasia. On the other hand, a fast onset may result from foreign bodies in the bronchus among individuals who have previously ingested them [14].

One of the leading causes of infant death, pulmonary hypoplasia, is frequently the result of underlying abnormalities, such as oligohydramnios, space-occupying lesions, chest wall deformities, and neuromuscular diseases. Different cases have varying degrees of severity; mild cases may not show symptoms in infancy but may later be susceptible to refractory respiratory infections, while severe cases may show signs of dyspnea, cyanosis, and respiratory failure. Imaging methods and differential diagnosis are essential to diagnose and treat the illness. Despite advancements, no proven treatments and related abnormalities typically complicate the results.

## Conclusions

This case report highlights the complex interplay between pulmonary hypoplasia and congenital heart defects, specifically in an 11-month-old child with left lung hypoplasia, a ventricular septal defect, and an absent left pulmonary artery. Despite the potential benefits of surgical intervention for the VSD, the child's parents opted against the procedure, underlining the critical role of parental decision-making in pediatric care. This case underscores the importance of early diagnosis and comprehensive management strategies to improve outcomes for children with similar congenital anomalies. Continued follow-up and supportive care are essential for monitoring the child's condition and addressing future complications.
